# High throughput generation and characterization of replication-competent clade C transmitter-founder simian human immunodeficiency viruses

**DOI:** 10.1371/journal.pone.0196942

**Published:** 2018-05-14

**Authors:** Debashis Dutta, Samuel Johnson, Alisha Dalal, Martin J. Deymier, Eric Hunter, Siddappa N. Byrareddy

**Affiliations:** 1 Department of Pharmacology and Experimental Neuroscience, University of Nebraska Medical Center, Omaha, Nebraska, United States of America; 2 Emory Vaccine Center, Emory University, Atlanta, Georgia, United States of America; 3 Department of Biochemistry and Molecular Biology, University of Nebraska Medical Center, Omaha, Nebraska, United States of America; 4 Department of Genetics, Cell Biology and Anatomy, University of Nebraska Medical Center, Omaha, Nebraska, United States of America; University of Pittsburgh Centre for Vaccine Research, UNITED STATES

## Abstract

Traditional restriction endonuclease-based cloning has been routinely used to generate replication-competent simian-human immunodeficiency viruses (SHIV) and simian tropic HIV (stHIV). This approach requires the existence of suitable restriction sites or the introduction of nucleotide changes to create them. Here, using an In-Fusion cloning technique that involves homologous recombination, we generated SHIVs and stHIVs based on epidemiologically linked clade C transmitted/founder HIV molecular clones from Zambia. Replacing *vif* from these HIV molecular clones with *vif* of SIVmac239 resulted in chimeric genomes used to generate infectious stHIV viruses. Likewise, exchanging HIV *env* genes and introducing N375 mutations to enhance macaque CD4 binding site and cloned into a SHIV_AD8-EO_ backbone. The generated SHIVs and stHIV were infectious in TZMbl and ZB5 cells, as well as macaque PBMCs. Therefore, this method can replace traditional methods and be a valuable tool for the rapid generation and testing of molecular clones of stHIV and SHIV based on primary clinical isolates will be valuable to generate rapid novel challenge viruses for HIV vaccine/cure studies.

## Introduction

Human immunodeficiency virus (HIV) is the major causative agent of acquired immunodeficiency syndrome (AIDS) in humans. Since there are several limitations in obtaining human tissues/samples other than blood, investigators use non-human primates (NHPs) as models to study HIV infection and pathogenesis. Several isolates of the simian immunodeficiency virus (SIV) cause an AIDS-like disease in NHPs. Since HIV does not infect NHPs, a chimeric virus, the simian-human immunodeficiency virus (SHIV) has been engineered to contain HIV genes such as Envelopes (Env) or Reverse transcriptase (RT) [1–4]. Although these initial SHIVs mimic the dynamics of infection in humans, they do not entirely recapitulate primary infection and pathogenesis. Therefore, there is currently an unmet need to develop infectious SHIVs using HIV Envs from field samples [1, 2, 4, 5]. HIV Env is highly and variably glycosylated and is considered the prime candidate for the evocation of the immune response [6–8]. Furthermore, various mutations in Env render HIV with efficient adaptability for infection [9].

Subtype C viruses are responsible for a majority of HIV infections worldwide. Therefore, generating SHIVs that carry Env of subtype C transmitted/founder viruses (T/F) will be a vital tool for the evaluation of vaccine-induced immunity and for *in vivo* studies of host-virus interaction [10]. The paucity of Env-containing SHIVs can be attributed to a number of issues including, the labor-intensive nature of deriving such constructs that previously required passage in NHP, and the potential for such constructs to be unstable and poorly infectious for NHP cells [3]. This is due to the species-specific interaction between the Env sequences that are required to bind to CD4, which serves as a primary receptor for cellular entry in the virus natural host. When comparing Env proteins, Env375 has been identified as the critical residue in binding to macaque CD4 by conferring greater amino acid volume in SIV compared to HIV, and substitution of serine from the HIV Env sequence with histidine or tryptophan from SHIV at this position conferred increased infectivity in macaque cells [11]. It should also be noted that before our novel technique was developed, successful cloning of Env-containing SHIVs was highly dependent upon the presence of unique restriction endonuclease (RE) sites. This method therefore relied on the presence of specific unique restriction sites, or the generation of specific RE sites for cloning through the insertion of new nucleotides modifying the amino acid sequence which could ultimately be affecting the expression or final protein structures in the virus. As we show here, the use of a recombinase-based cloning system obviates the need for specific restriction sites and allows optimal junction points in the construct to be utilized.

Earlier studies demonstrated that the failure of HIV to replicate in rhesus macaque (RM) cells *in vitro* could be overcome by rendering the virus resistant to the TRIM5α and APOBEC3 proteins [12–14]. Pig-tailed macaques lack a TRIM5α protein; instead, they express TRIMCyp, which does not restrict HIV replication. Consequently, only the *vif* gene needs to be replaced to overcome the inhibition mediated by APOBEC3G. This concept was demonstrated recently in which HIV *vif* was substituted and replaced with either SIVmac239 or HIV-2 *vif*, resulting in chimeric viruses that were replication competent in pig-tailed macaques [15]. This finding opens up the possibility that with minimal engineering, HIV can be adapted to replicate in macaques.

To overcome these limitations and to rapidly generate replication competent SHIV/stHIVs, we used a hurdle-free homologous recombination method [16, 17]. We show that this method facilitates the generation of highly infectious replication-comptent molecular clones encoding the *env* gene from T/F viruses that will be useful in vaccine/cure studies using macaques as models.

## Materials and methods

### Ethics statement

Rhesus monkeys (*Macaca mulatta*) were utilized in this study were procured from New Iberia Primate Research Center, New Iberia, LA, USA. All animals were housed on cages with 6.0 ft^2^ floor space modules and maintained at the Department of Comparative Medicine at the University of Nebraska Medical Center (UNMC) in accordance with the rules and regulations of the Committee on the Care and Use of Laboratory Animal Resources, and according to the guidelines of the Committee on the Care and Use of Laboratory Animals of the Institute of Laboratory Animal Resources, National Research Council and the Department of Health and Human Services guidelines titled Guide for the Care and Use of Laboratory Animals. All protocols and procedures were performed under approval of the UNMC Institutional Animal Care and Use Committee according to the National Institute of Health guidelines. The animals were fed a monkey diet (Purina, Wilkes-Barre, PA) supplemented daily with fresh fruits or vegetables and wate with regular light cycle of 12 hours light and 12 hours dark. Additional social enrichment, including the delivery of appropriate safe toys, was provided and overseen by the UNMC enrichment staff. Animal health was monitored daily and recorded by the animal care staff and veterinary personnel, available 24 hours a day and 7 days a week. Monkeys were caged in socially compatible same sex pairs to facilitate their well-being and social enhancement. The UNMC primate Housing facility has been fully accredited by the Association for Assessment and Accreditation of Laboratory Animal Care International. The studies reported herein were performed under IACUC protocol 15-072 Transmitted/ Founder SHIV Macaque Model; 15-073 Role of HIV Env glycosylation in mucosal transmission which was reviewed and approved by the UNMC IACUC. In addition, all experiments were reviewed and approved by the UNMC biosafety review Committee prior to initiation of the studies.

### Source of blood samples

There is no intervention involved in the studies conducted herein. Only heparin/EDTA blood samples were collected from monkeys anesthetized with Ketamine (10mg/kg IM). All the animals were negative for SIV, simian T-cell lymphotropic virus, and simian retrovirus.

### In-Fusion cloning of HIV env in pSHIV_AD8-EO_

Full length HIV molecular clones were generated using infectious samples from Zambia previously described [10, 18]. We utilized *env* genes from a set of infectious HIV molecular clones obtained from the transmitting source partner (TSP clones) derived from a set of subtype C epidemiologically-linked transmission pairs from the Zambia-Emory HIV Research Project (ZEHRP) in collaboration with Dr. Eric Hunter. The details of generation of infectious HIV viruses and its characterization were published previously [10, 18]. *Env* gene from these molecular clones were used for the generation of T/F SHIVs. These HIV molecular clones (S1 Table) were first validated for sequence information by sanger sequencing then transiently transfected for virus generation. Their infectivity was verified using the TZMbl cells. *Env* genes (full length, *tat-rav-vpu-env-tat-rav* and truncated, minimal *env* region without regulatory *tat-rav-vpu*) were amplified from respective HIV clones. Forward and reverse primers were designed to carry a 15 base pair region (S2 Table). This region was homologous to that of reverse and forward primers used for vector backbone and linearized by inverse PCR. In brief, PCR was carried out using CloneAmp HIFi PCR premix (Takara Bio, CA, USA), Inverse PCR condition 98^0^C-2’, (98^0^C-25”, 60^0^C-30”, 72^0^C-5’30”)_24_ and 4^0^C- 10’ for vector linearization. Conditions for *env* amplifications from HIV Zambian infectious molecular clones was 98^0^C-2’, (98^0^C-25”, 60^0^C-30”, 72^0^C-2’30”)_24_ and 4^0^C- 10’. Finally, the linearized vector backbone and *env* were mixed at an appropriate ratio, In-Fusion mix and incubated at a specified temperature (37^0^C-15’, 50^0^C-15’ and 4^0^C-10’) to allow homologous recombination. Thereafter, this mix was transformed using Stellar Competent Cells as per as the manufacture’s instructions (Takara Bio, CA, USA). Colonies were screened after a 16-hour incubation at 30^0^C. Clones were confirmed by restriction digestion and validated by Sanger sequencing.

### In-Fusion cloning for the generation of stHIV

For the generation of stHIV, highly infectious molecular clones of HIV were chosen (S1 Table) as described previously [10, 18]. To overcome the APOBEC restriction, *vif* from these HIV molecular clones were replaced by SIVmac239 *vif* [12–14]. The full-length HIV molecular clones were linearized by inverse PCR except the *vif* portion. *vif* was amplified from pSHIV_AD8-EO_ using reverse and forward primer carrying 15 bp homologous to the forward and reverse primers used for vector linearization (S2 Table). Both the PCR products, vector backbone (HIV), and insert-*vif* were purified from agarose gel and subjected for In-Fusion based homologous recombination. The In-Fusion mix with appropriately mixed vector and insert was transformed into Stellar Competent Cells (Takara Bio, CA, USA). Colonies were screened after 16-hrs incubation at 30^0^C and clones were confirmed by restriction digestion and validated by Sanger sequencing.

### In-Fusion site-directed mutagenesis for Env375 mutation

To replace Env375 serine by other amino acids to provide SHIV and stHIV better entry into macaque CD4 cells, In-Fusion site-directed mutagenesis was used. Briefly, primer was designed from the region where mutation is to be introduced (S3 Table). To facilitate homologous recombination, both forward and reverse primers were designed in such a way to provide 15bp homologous regions in the 5 prime of each. The position of mutation was identified as Env375. The nucleotides were replaced in the primer in the middle of the overlap region to encode desired amino acid replacing serine. PCR condition used was 98^0^C-2’, (98^0^C-25”, 60^0^C-30”, 72^0^C-3’50”)_24_ and 4^0^C- 10’ using CloneAmp HIFi PCR Premix (Takara Bio, CA, USA).

### Generation of virus stocks and infectivity

Cloned confirmed chimeric SHIVs and stHIVs and their Env mutated version were transiently transfected into HEK293T cells using ietPRIME transfection reagent (Polyplus Transfection, NY, USA). Cell-free viral supernatants were harvested 72 hrs post-incubation and tested for their infectivity using recombinant TZMbl cells. Briefly, viruses are incubated with TZMbl cells for 48 hrs; after this, Luciferase assay (using britelite plus reagent; PerkinElmer, Boston, USA) was carried out to measure the infectivity of each virus. Relative luminescence produced by each virus was measured through 1:5-fold serial dilutions and then plotted [5, 19]. Finally TCID_50_ was calclulated as described previously [20]. The infectivity of generated viruses was quantified by incubating with recombinant TZMbl cells followed by luciferase assay We have measured the infectivity of HEK 293T generated SHIVs using TZMbl cells and β-gal staining followed by counting blue cells [21]. Briefly 2,000 TZMbl cells were seeded in 24 well tissue culture plate. 5-fold serially diluted viruses were infected for limiting dilutions in duplicate. After 2 hrs of infection cells were washed and further incubated for 48 hrs. Post 48 hrs incubation cells were washed and stained with β-gal, blue cells were counted and infectious units were calculated [21].

### *In vitro* replication of SHIVs in PBMCs

PBMCs were isolated from naïve monkeys using polyprep (Invitrogen, NY, USA) and cultured in RPMI supplemented with 20% FBS. PBMCs were stimulated with concovalin A (25ug/ml; Sigma-Aldrich) on day zero, after which the cells were cultured for three days with human recombinant IL-2 (20U/ml). After 72 hrs stimulation, PBMC were spinoculated by adding 200TCID_50_ of each SHIV generated by transfecting HEK293T cells [4, 22]. For each infection 2x10^6^ PBMC were used in 24 well plate. After infection celles were washed twice and cultured in RPMI supplemented by 20% FBS and IL-2 (20U/ml). 200ul supernatants from cultured cells were collected at two day intervals up to day 21 from infection, and was replaced by fresh medium with IL-2. Replication kinetics of SHIVs in macaque PBMC were ploted by measuring p27 levels using P27 ELISA kits (ABL inc, Rockville, USA) throughout the infection upto day 20.

### Sequencing

All amplicons and clones were directly Sanger sequenced at University of Nebraska Medical Center DNA sequencing core, aligned, and analyzed as has been described previously [1].

### Virus entry assay

To monitor differences in entry of viruses due to Env mutations, TZMbl cells were used, which expresses human CD4 and CCR5, receptor and co-receptor respectively as described previously [11, 19, 21, 23]. Briefly, wild-type and mutated viruses (200TCID_50_ and 5-fold serially diluted) were used for the infection of TZMbl cells in duplicate in the presence of DEAE Dextran (10μg/ml) and were washed after 2 hrs of infection. After 48 hrs of infection, luciferase activity was read using britelite plus reagent (PerkinElmer, Boston, USA) to determine the relative viral entry into cells. Similarly, viral entry was also carried out using ZB5 cells [11], which expresses rhCD4 and rhCCR5 to the comparable level of huCD4 and huCCR5 in TZMbl cells.

## Results

### Generation of SHIV by cloning of HIV envelope in SHIV_AD8-EO_

**Fig 1:**
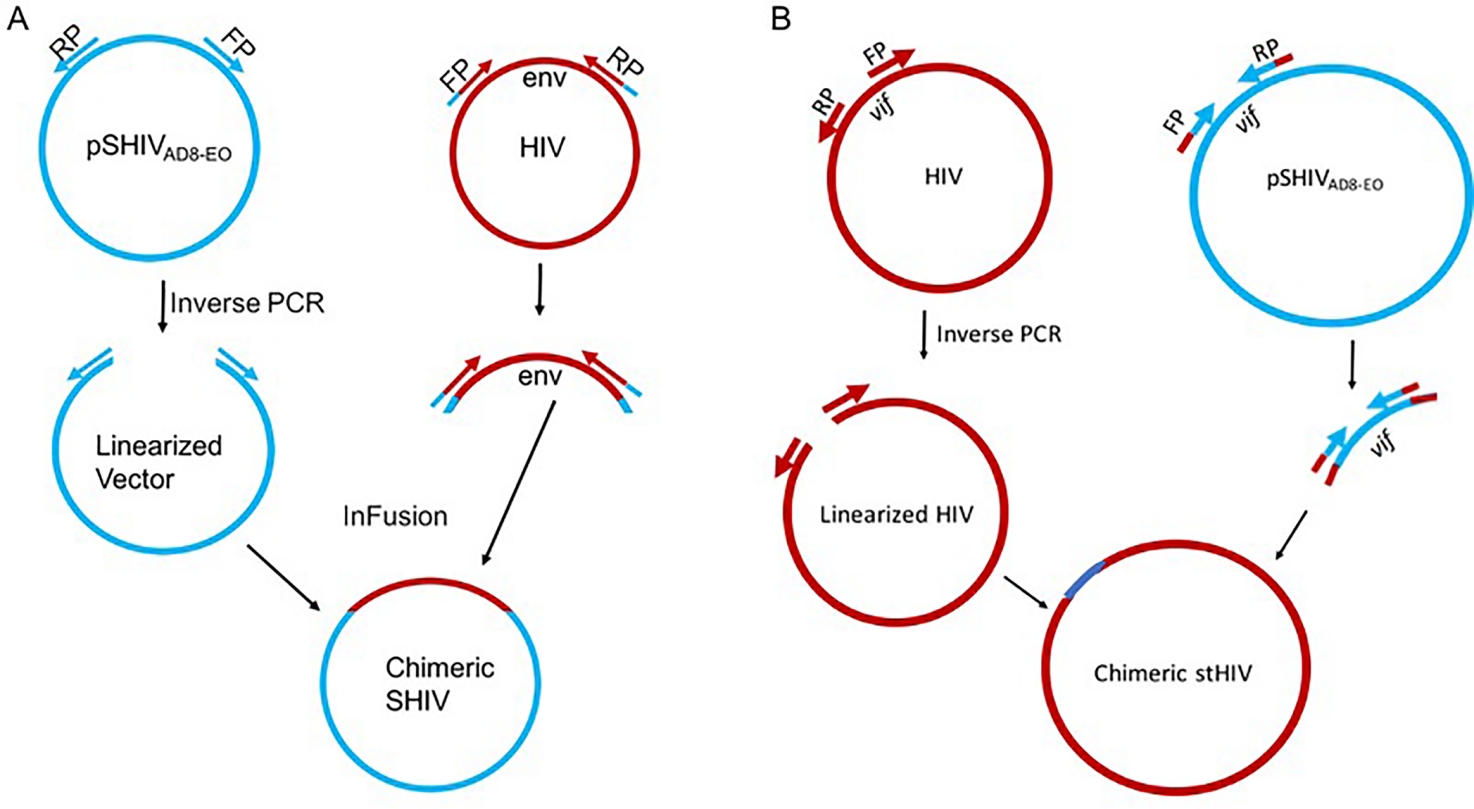
**Schematic representation of the In-fusion cloning steps for the generation of (A) Simian Human Immunodeficiency Virus (SHIV) and (B) Simian tropic Human Immunodeficiency Virus (stHIV).** Inverese PCR was used for the linearization of the vector backbone, **pSHIV**_**AD8-EO.**_ **HIV** *env* gene specific primers were used for the amplification of the each *env* from specific HIV infectious molecular clones as described previously.

**Fig 2:**
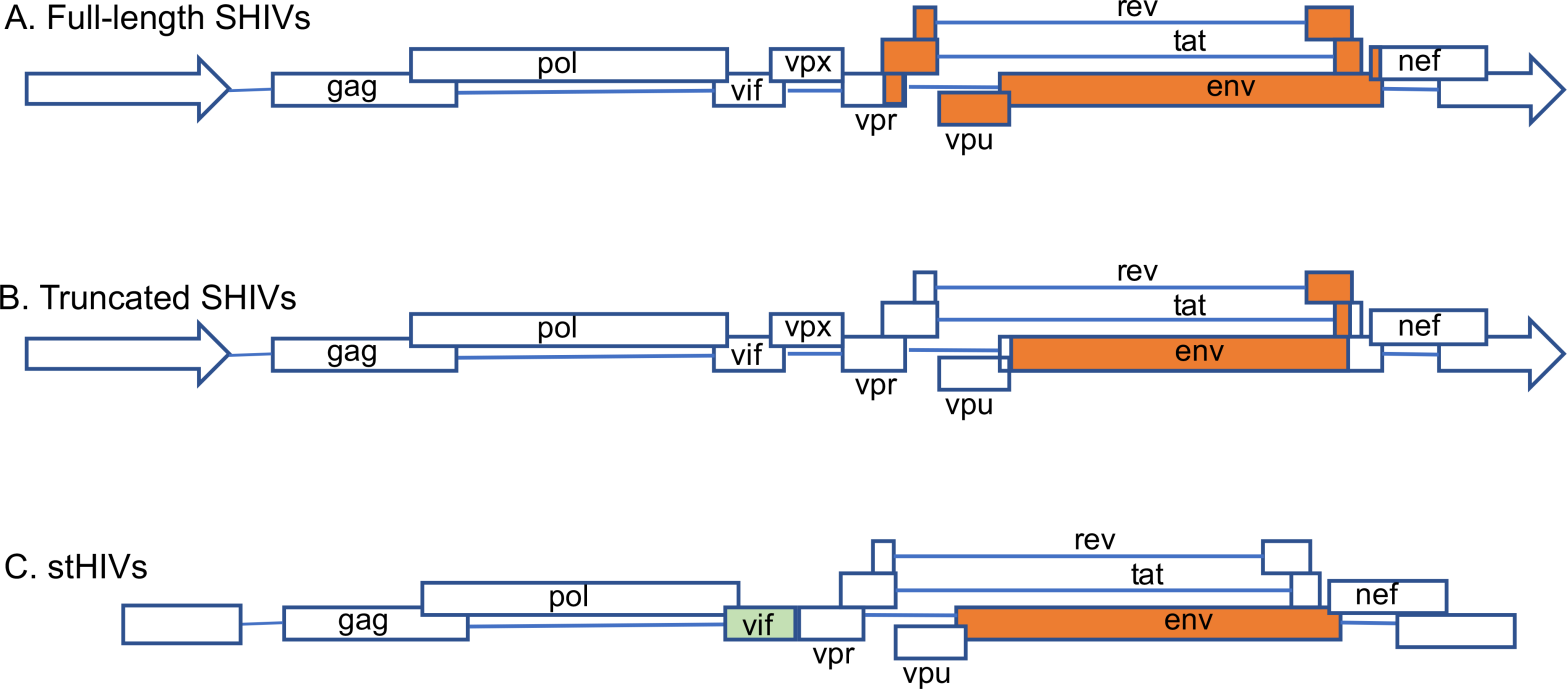
Genomic organization depicting portions of *env* used in the construction of (A) SHIVs with full length clade C HIV Zambian T/F *env* genes (B) SHIVs with truncated *env* genes and (C) stHIV, where *vif* of HIV molecular clones were replaced by SIV mac239 *vif*.

**Fig 3:**
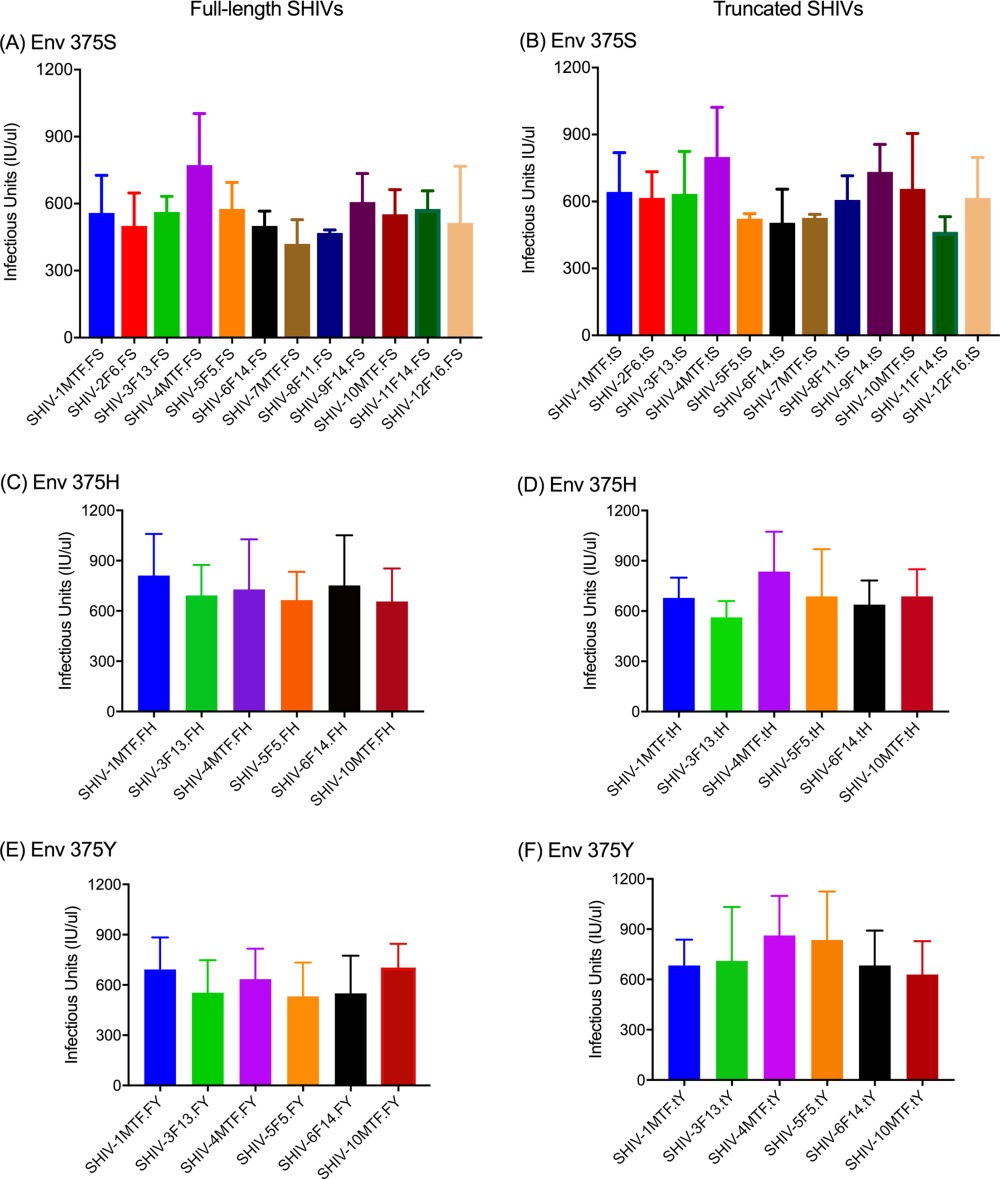
Infectivity of various SHIVs were measured using β-gal assay. TZMbl cells were infected with 200TCID_50_ of 293T cell generated viral supernatant using standard TZMbl assays as described previously. After 72-hrs post infection β-gal assay was measured and Infectious units are calculated. Full-length SHIVs *env* 375S (panel A) with *env* 375H (panel C) and *env* 375Y (panel E). Similarly, truncated SHIVs env 375S (panel B) with env 375H (panel D) and env 375Y (panel F).

**Fig 4:**
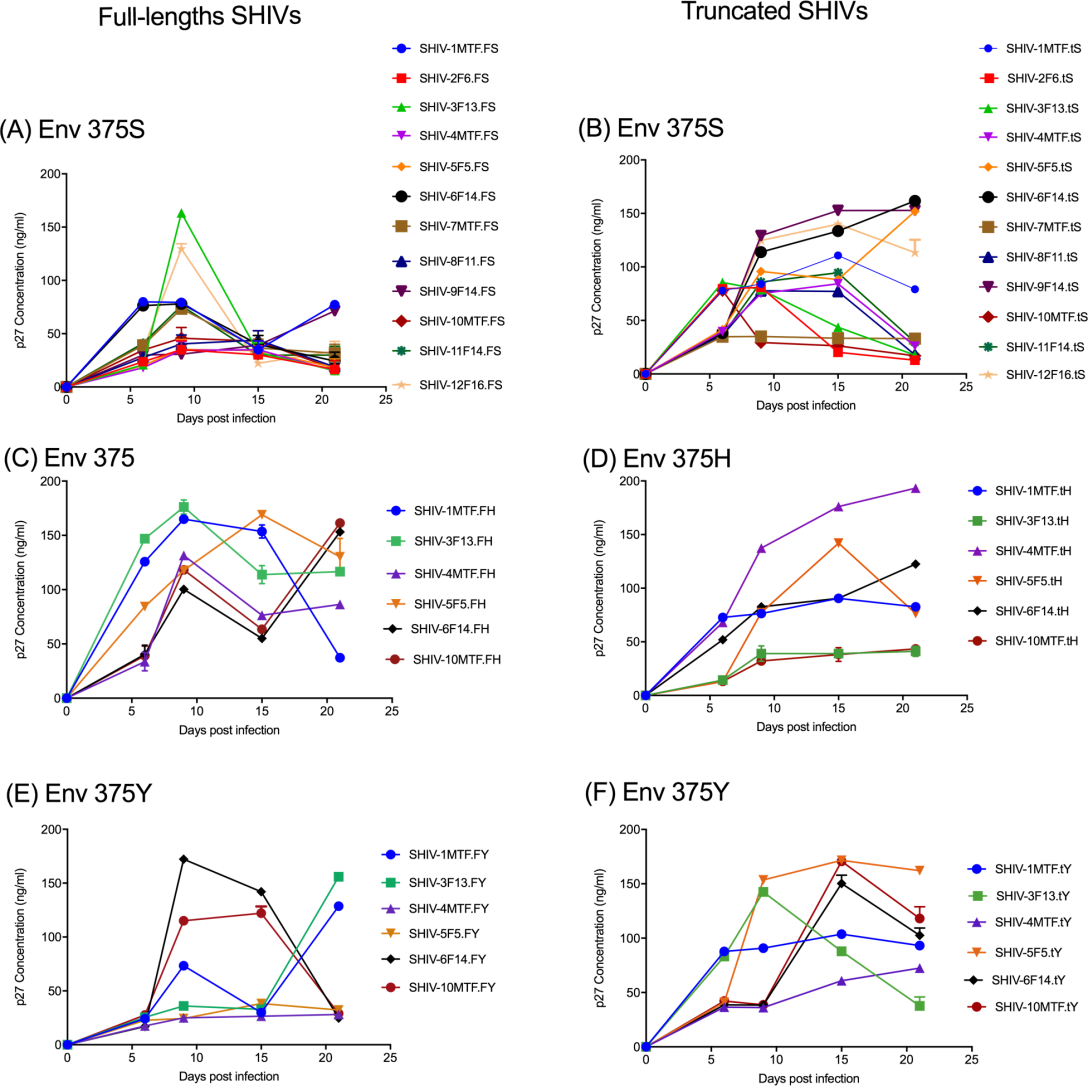
Replication kinetics of newly generated SHIVs in rhesus macaque PBMCs. Pooled PBMC from randomly selected naive RM donors were stimulated with concanavalin A (Con-A) for 48-72hrs washed and infected with 200TCID_50_ of various SHIVs as described in the method. Culture supernatents were harvested every three days upto 20-25 days and SIV gag protein (P27) were measured using P27 ELISA kits. Left panel represents a replication kinetics of full-length SHIVs as well as S375H/Y mutation and right panel represents truncated SHIVs and S375H/Y mutation.

We successfully cloned the HIV *env* from infectious molecular clones of HIV for the generation of SHIV without the use of RE, which has been the traditional method. We used the commercially available In-Fusion kit (Clontech, Mountain View, CA) that works on the principle of recombination between homologous regions in two DNA sequences. For SHIV generation, the SIVmac293 based vector backbone SHIV_AD8-EO_ was used [24]. The vector backbone, except for the *env*, was linearized by inverse PCR, using forward and reverse primers (S2 Table) that provide 15bp sequences at each end for homologous recombination to the insert (*env* PCR products of HIV clones). The *env* genes of 12 HIV molecular clones (4 TF and 8 non-transmitted variants (NT); S1 Table) from Zambia [18] were amplified with the addition of 15 nucleotides in both forward as well as reverse primers to provide vector sequences that allow homologous recombination partners for the vector pSHIV_AD8-EO_ (Fig 1A). Both, the linearized vector backbone SHIV_AD8-EO_ and the amplified *env* were incubated with In-Fusion mix at a temperature specified by the manufacturer and transformed into Stellar Competent Cells. SHIVs generated using the HIV *env* genes were further divided into two categories: full-length SHIVs carrying *tat-rev-vpu-env-tat-rev* from HIV and truncated SHIVs carrying a minimal HIV *env*-region (Fig 2A and 2B) (S1 Table). The recombinant SHIVs obtained using homologous recombination were infectious and replication competent. Infectivity was determined by transient transfection of 293T cells followed by the testing of supernatant fluids using the TZMbl cells by β-gal staining and luciferase readings (Fig 3 and S1 Fig). To test the replication competence of newly developed SHIVs we used randomly selected naïve macaque PBMCs. The cell supernatant fluids from the 293T cells were used to infect *in vitro* activated rhesus peripheral blood mononuclear cells (PBMC). As presented in Fig 4 the newly developed SHIVs were found to be replication comptent with peak P27 protein detected in the culture supernatants between 10-15 days for full length viruses and upto 20 days for truncated SHIVs. Overal, truncated SHIVs were found to be more replication comptent as compared to full-length SHIVs. Cultures were performed in the presence/absence of AZT as controls in the assay.

### Generation of simian tropic HIV (stHIV) by replacing HIV *vif* by SIVmac239 *vif*

**Fig 5:**
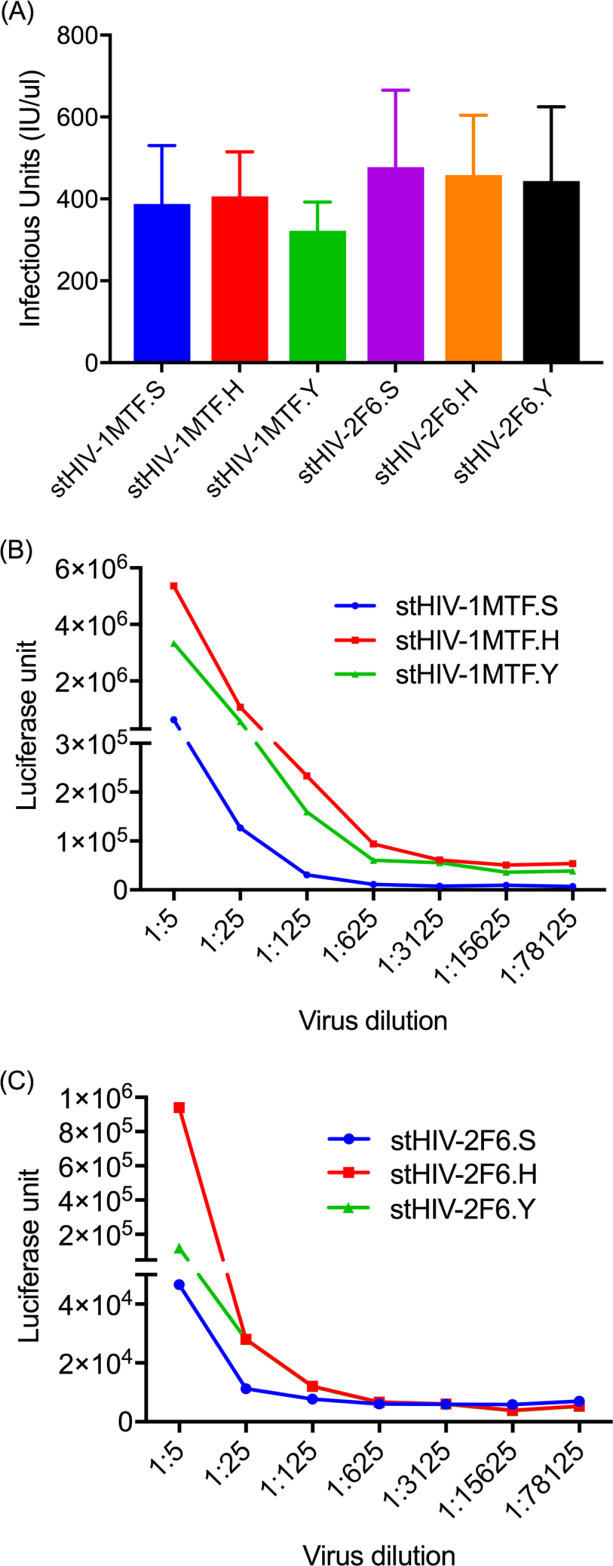
Infectivity of various stHIVs were measured in TZmbl cells using β-gal and luciferase assays. HEK 293T cells supernatant of newly generated stHIV with Env375H or Env 375Y viruses were infected (200TCID_50_) with TZMbl cells and after 48-72 hr (A) Infectious units were calculated by β-gal staining to confirm the infectivity of the viruses (B and C) Luciferase activity was measured after adding britelite plus reagent.

Earlier, several studies generated stHIV by using traditional cloning and was successful in generating infectious stHIV clones [12–14]. In order to demonstrate the utility of In-Fusion cloning in generation of stHIV we replaced the *vif* of HIV by SIV to overcome the APOBEC restriction factor (Figs 1B and 2C). The entire HIV genome, except *vif*, was amplified using inverse PCR. Similarly, we also amplified SIV mac293 *vif* from SHIV_AD8-EO_ (Figs 1B and 2C). Both linearized HIV and amplified *vif* were incubated with In-Fusion mix at the specific temperature outlined in the methods section and transformed into Stellar Competent Cells. The recombinants clones were confirmed by restriction digestion and by sanger sequencing. The newly obtained recombinant clones were transfected into 293T cells and supernatant were tested in TZMbl cells. As shown in Fig 5, newly generated stHIV clones were infectious and replication comptent as measured by β-gal staining for the infevtivity of cells and luciferase acitivity in the supernatent.

### Env CD4 binding region mutation using In-Fusion site-directed mutagenesis

It has recently been discovered that the failure of HIV Env to bind to rhesus CD4 molecules is the basis for the failure of SHIV’s to infect and replicate in rhesus macaques. The identification of the crucial residue hampering CD4 binding both in CD4 as well as in Env prompted us to exploit this knowledge [11, 25, 26]. The conclusion that Env 375 plays a critical role in CD4 binding were drawn based on the original residue as present in SIV and mutations selected by nature in the circulating forms of HIV-1 [17, 25]. Therefore, we engineered these minimal mutations into our newly generated SHIVs and stHIVs. We have replaced Env375S with H and Y (Serine with Histidine or Tyrosine) in both SHIV and stHIV using In-Fusion based novel homologous recombination technology for site-directed mutagenesis. The In-Fusion site-directed mutagenesis is efficient, reliable, and resulted in > 99% success in inserting the desired mutation (S1 Table). SHIV clones tested showed replication competency and in some cases demonstrated enhanced infectivity as compared to the wild type in TZMbl cells (Fig 3C–3F; S1 Fig) and macaque PBMCs (Fig 4C–4F) as reported previously [17, 25]. As might be expected few difference were observed between the modified and WT stHIV in TZMbl cells (Fig 5B and 5C).

### Effect on infectivity of SHIVs due to Env375 mutation

**Fig 6:**
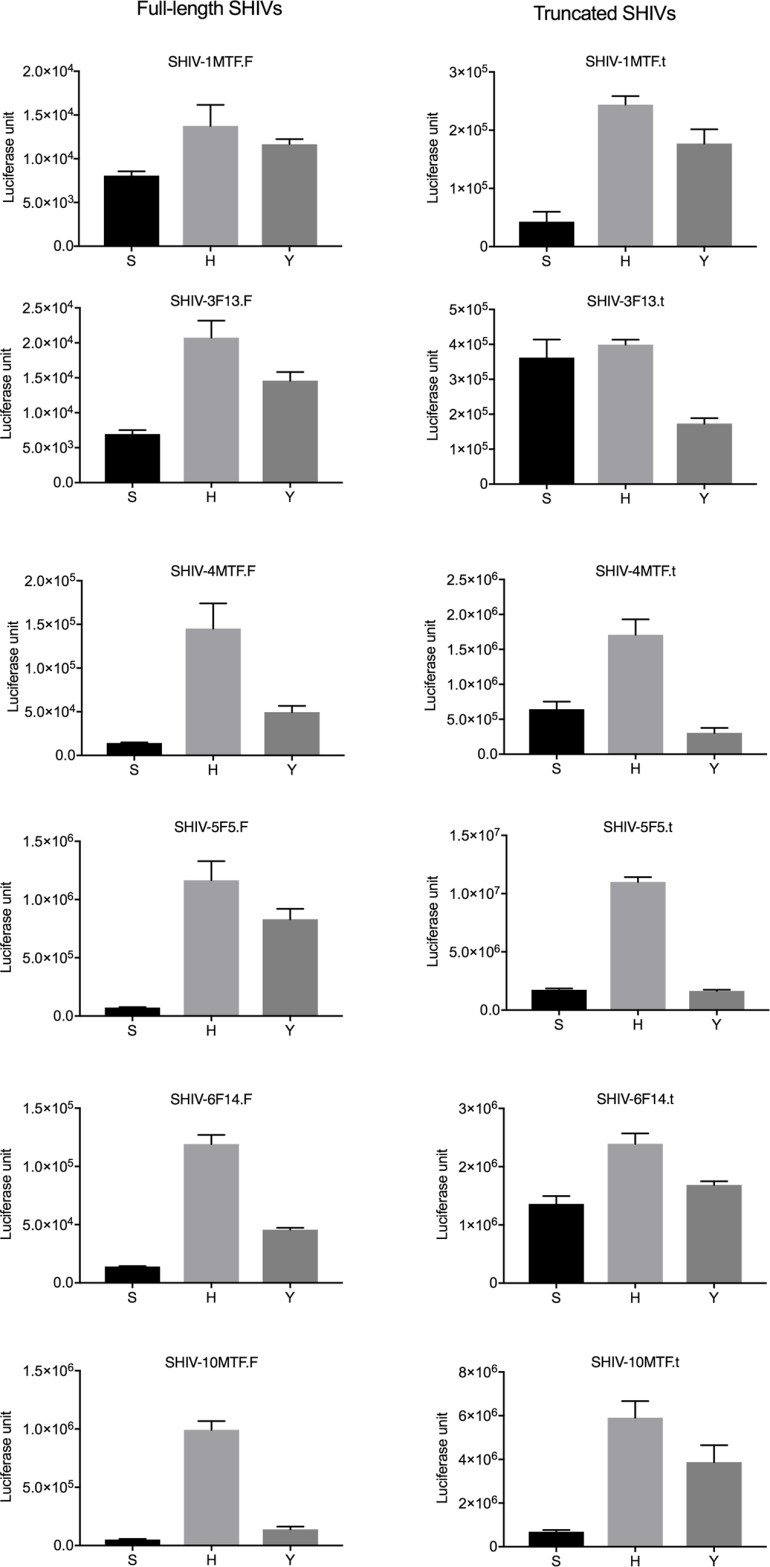
Virus entry assay in ZB5 cells. Selected full-length and truncated SHIVs with either Env 375S (wt with serine) or Env 375H (mutated to Histidine) or Env 375Y (mutated to Tyrosine) viruses were tested for their ability to enter cells using ZB5 cells which expresses rhesus CD4 and CCR5 at equal level to human CD4 and CCR5 in TZMbl cells. Cells were seeded at 1 × 10^4^ per well in 96-well plates and incubated 200TCID_50_ of virus for 5 hrs and virus was removed washed cells and replaced with fresh complete medium. After 48 hrs of post-infection britelite plus reagent was added to measure luciferase activity. As a control cells alone without infection were used to correct for background luciferase activity.

It is evident from the recent reports that the Env375 mutation is crucial for viral entry into CD4+T cells [11, 17]. To validate if the increased infectivity observed with the mutated SHIVs constructed here was due to enhanced viral entry, we performed viral entry with ZB5 cells using similar protocols and procedures described previously [11]. The ZB5 cells were engineered to express macaque CD4 and CCR5 [11]. As described in the Fig 6 mutation of Env375 from Serine (S) to Tyrosine (Y) increased viral entry into cells, and a further enhancement was observed with a Histidine (H) mutation in most of the SHIVs tested (Fig 6). Ongoing studies will reveal whether these clones will replicate optimally in rhesus macaques.

## Discussion

There is a high urgency for a more optimal animal model for HIV research that would facilitate vaccine and cure development. Generating a SHIV with high replication competency would remedy this need and can be achieved by cloning the HIV *env* gene into the SIVmac239 backbone. Currently, several such SIV backbones have been used for SHIV generation, and in a recent finding, it was demonstrated that the backbone plays a minimal role in the degree of infectivity of SHIVs [11]. In the present study, we used the SHIV_AD8-EO_ backbone. This is already adapted to macaques and is shown to be highly infectious and leads to sustained viremia following reaching plasma viral load set points in Indian origin rhesus macaques [24]. Until now, conventional restriction-based methodology has been the sole method for this type of cloning [1, 11, 22]. The main disadvantage of this conventional methodology for SHIV cloning is that it is dependent on the presence or generation of exclusive restriction sites. To overcome this limitation, we successfully used the recently developed homologous recombination-based In-Fusion cloning technique, which does not need any restriction sites and has fewer limitations than restriction-ligation cloning, which rely on such sites for cloning. This method is simple and accurate as evident from genome manipulation of other organisms in adition to HIV full-length molecular clones [18, 27]. Therefore, we used this method to generate SHIVs and stHIVs, including the introduction of point mutations to enhance CD4 binding [11, 26, 28]. The In-Fusion cloning works on the principle of homologous recombination using only a 15 base-pair homologous region [27, 29]. For SHIV cloning, the vector backbone of pSHIV_AD8-EO_ was linearized except for the *env* using inverse PCR. The *env* gene was excluded from the linearized vector backbone by designing primers flanking the gene. The respective HIV *env* genes were amplified using specific primers for each of the available molecular clones of HIV by PCR. Similar methodology was used as described in Fig 1B for stHIV cloning. The other major obstacle for SHIV cloning has been the overlapping of *env* with regulatory genes. This includes *vpu* that is exclusive to HIV and absent in SIV; similarly, *vpx* is present in SIV and absent in HIV. In addition, *vpr* positions differ in SIV and HIV, and the env/nef overlap is critical for SHIV replication. Therefore, the differences in the architecture of genes in both the HIV and SIV genomes present a challenge in placing one gene on the backbone for the generation of infectious, chimeric SHIVs. By using the homologous recombination method, we have overcome such technical challenges and generated SHIVs encoding either HIV or SIV accessory genes.

Previous findings have suggested that a mutation at Env375 is critical for virus entry and infectivity [11, 17]. Some of these mutations were naturally selected in circulating forms of HIV and have shown resistance for the entry inhibitors [25]. Furthermore, it has been demonstrated that most circulating forms of HIV depend on the histidine residue for establishing initial infection. We therefore generated these mutations in the T/F viruses and found that these mutations increased infectivity by facilitating viral entry in rhesus cells [11]. An entry assay on ZB5 cells clearly demonstrated enhancement of virus entry due to the mutation of serine (S) to Tyrosine (Y) and Histidine (H). We propose that the In-Fusion based cloning of HIV *env* into an SIV backbone described here, will open up avenues for faster and easier generation of SHIV and stHIV thereby eliminating the need for restriction endonucleases and ligase. Therefore, this technique can be easily adapted for high-throughput generation of SHIV/stHIV from primary HIV isolates.

Unlike traditional cloning using restriction endonucleases and other enzymes, we herein demonstrated the successful use of homologous recombination based on the In-Fusion cloning technique adapted for the rapid generation of SHIV and stHIV. Using this In-Fusion cloning, we generated a number of SHIVs, stHIVs, and introduced point mutations at S375 position on several *env* genes. These SHIVs/stHIVs were replication competent in TZMbl cells and primary cultures of macaque PBMC suggesting that replication kinetics of newly generated viruses were comparable to traditionally generated viruses by others and our group previously [1, 4, 11, 30–32]. Using this method, it is evident that generation of SHIVs and stHIV is rapid and scalable for the generation of a large number of SHIVs/stHIVs as tools for vaccine or cure studies and to test number of broadly neutralizing monoclonal antibodies in passive immunization studies. On-going *in vivo* studies will determine whether these SHIVs/stHIVs are replication competent, maintain viral set points, and progress to disease.

## Supporting information

S1 Fig Replication kinetics of newly generated SHIVs in TZMbl cells. TZMbl cells encode the luciferase gene under the control of the HIV-1 promoter; both CD4 and CCR5 are also expressed on the cell surface. A total of 6,000 cells/well were seeded in 96- well plates. Serial 5-fold dilutions of various viruses were prepared in triplicates in another plate and 15 μg/ml DEAE-Dextran (final concentration) solution was added to all wells and the entire mixture was transferred into the 96-well flat–bottom plate with the seeded TZMbl cells. The next day, medium was replaced with fresh medium and incubated another 24h-48h. Britelite plus reagent was added to the plate the following day and luciferase activity was measured. Left panel represents (A,C and E) for wild type full length SHIVs, Env 375H, and Env 375Y. Right panel represent (B, D and F) truncated SHIVs wild type and Env 375H, and Env 375Y.
(TIFF)

S1 Table List of HIV Infectious Molecular Clones (IMCs) and newly generated constructed SHIVs and stHIVs. (DOCX)

S2 Table Primers used for the construction of full-length, truncated SHIVs and stHIVs. (DOCX)

S3 Table Primers used for generation of env 375H and Y mutation, Same set of forward and reverse primer were used to generate mutation at the position Env 375 for full-length and truncated SHIVs. (DOCX)
