# Supplementary figures and images for: High throughput generation and characterization of replication-competent clade C transmitter-founder simian human immunodeficiency viruses
Source: PLoS One. 2018 May 14;13(5):e0196942. doi: 10.1371/journal.pone.0196942 (PMC5951672; doi:10.1371/journal.pone.0196942)

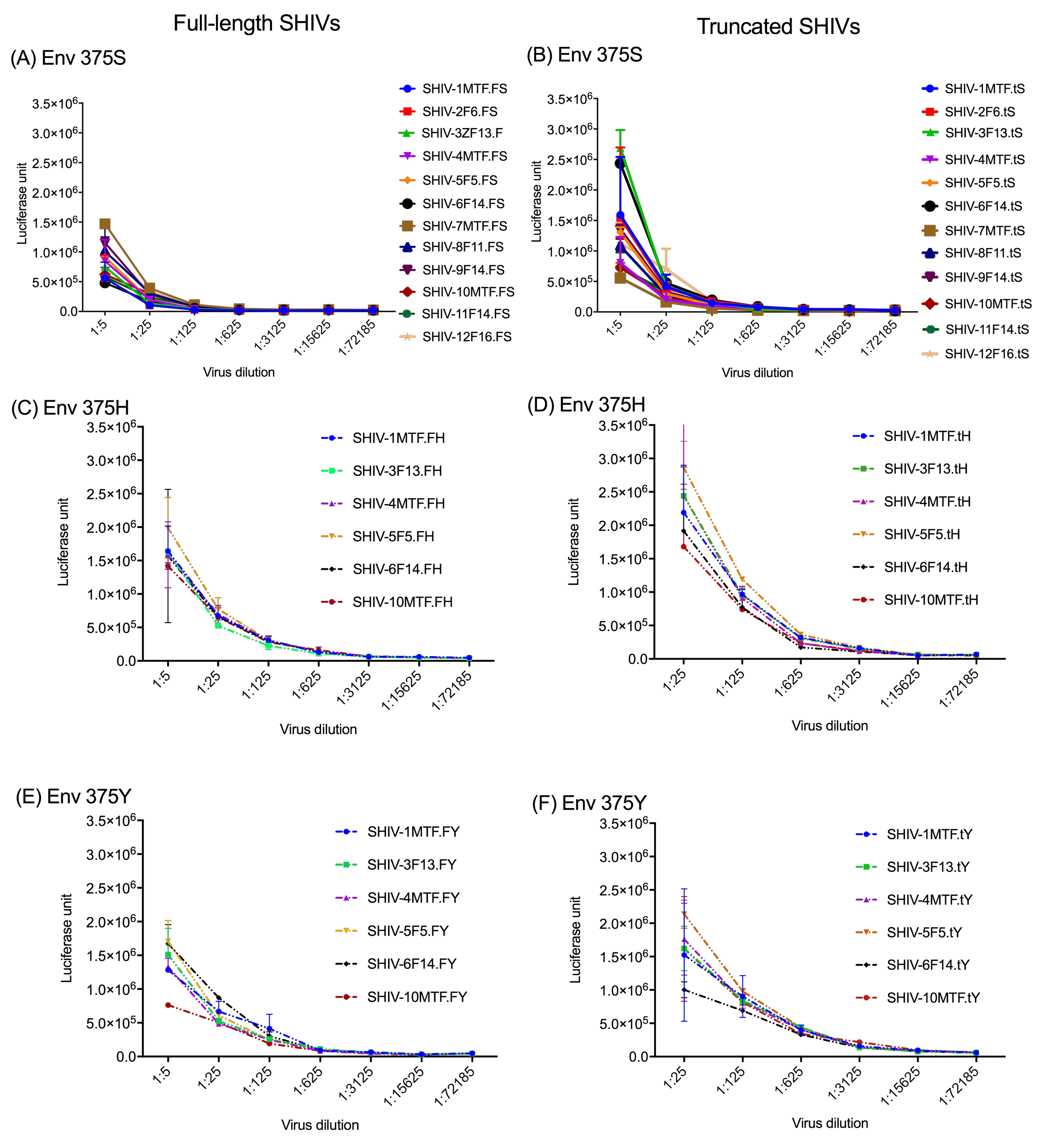

Supplement: S1 Fig — TZMbl cells encode the luciferase gene under the control of the HIV-1 promoter; both CD4 and CCR5 are also expressed on the cell surface. A total of 6,000 cells/well were seeded in 96- well plates. Serial 5-fold dilutions of various viruses were prepared in triplicates in another plate and 15 μg/ml DEAE-Dextran (final concentration) solution was added to all wells and the entire mixture was transferred into the 96-well flat–bottom plate with the seeded TZMbl cells. The next day, medium was replaced with fresh medium and incubated another 24h-48h. Britelite plus reagent was added to the plate the following day and luciferase activity was measured. Left panel represents (A,C and E) for wild type full length SHIVs, Env 375H, and Env 375Y. Right panel represent (B, D and F) truncated SHIVs wild type and Env 375H, and Env 375Y. (TIFF) [file pone.0196942.s001.tiff]
