# Supplementary material for: High throughput generation and characterization of replication-competent clade C transmitter-founder simian human immunodeficiency viruses
Source: PLoS One. 2018 May 14;13(5):e0196942. doi: 10.1371/journal.pone.0196942 (PMC5951672; doi:10.1371/journal.pone.0196942)
Supplement: S1 Table — (DOCX) [file pone.0196942.s002.docx]

Table S1. List of HIV Infectious Molecular Clones (IMCs) and constructed SHIVs and stHIVs

| Sr.No | Zambian HIV clones | SHIV.F  S375 | SHIV.F  H375 | SHIV.F  Y375 | SHIV.t  S375 | | SHIV.t  H375 | SHIV.t  Y375 |
| --- | --- | --- | --- | --- | --- | --- | --- | --- |
| 1 | Z331M TF | SHIV-1MTF.FS | SHIV-1MTF.FH | SHIV-1MTF.FY | SHIV-1MTF.tS | | SHIV-1MTF.tH | SHIV-1MTF.tY |
| 2 | Z331F 6 | SHIV-2F6.FS |  |  | SHIV-2F6.tS | |  |  |
| 3 | Z331F 13 | SHIV-3F13.FS | SHIV-3F13.FH | SHIV-3F13.FY | SHIV-3F13.tS | | SHIV-3F13.tH | SHIV-3F13.tY |
| 4 | Z3618M TF | SHIV-4MTF.FS | SHIV-4MTF.FH | SHIV-4MTF.FY | | SHIV-4MTF.tS | SHIV-4MTF.tH | SHIV-4MTF.tY |
| 5 | Z3618F 5 | SHIV-5F5.FS | SHIV-5F5.FH | SHIV-5F5.FY | SHIV-5F5.tS | | SHIV-5F5.tH | SHIV-5F5.tY |
| 6 | Z3618F14 | SHIV-6F14.FS | SHIV-6F14.FH | SHIV-6F14.FY | SHIV-6F14.tS | | SHIV-6F14.tH | SHIV/-6F14.tY |
| 7 | Z3678M TF | SHIV-7MTF.FS |  |  | SHIV-7MTF.tS | |  |  |
| 8 | Z3678F 11 | SHIV-8F11.FS |  |  | SHIV-8F11.tS | |  |  |
| 9 | Z3678F 14 | SHIV-9F14.FS |  |  | SHIV-9F14.tS | |  |  |
| 10 | Z4248M TF | SHIV-10MTF.FS | SHIV-10MTF.FH | SHIV-10MTF.FY | SHIV-10MTF.tS | | SHIV-10MTF.tH | SHIV-10MTF.tY |
| 11 | Z4248F 14 | SHIV-11F14.FS |  |  | SHIV-11F14.tS | |  |  |
| 12 | Z4248F 16 | SHIV-12F16.FS |  |  | SHIV-12F16.tS | |  |  |
|  |  | stHIV  S375 | stHIV  H375 | stHIV  Y375 |  | |  |  |
| 13 | Z331M TF | StHIV-1MTF.S | StHIV-1MTF.H | StHIV-1MTF.Y |  | |  |  |
| 14 | Z331F 6 | StHIV-2F6.S | StHIV-2F6.H | StHIV-2F6.Y |  | |  |  |
