# Supplementary material for: High throughput generation and characterization of replication-competent clade C transmitter-founder simian human immunodeficiency viruses
Source: PLoS One. 2018 May 14;13(5):e0196942. doi: 10.1371/journal.pone.0196942 (PMC5951672; doi:10.1371/journal.pone.0196942)
Supplement: S2 Table — (DOCX) [file pone.0196942.s003.docx]

Table S2. Primers used for the construction of Full-length, truncated SHIVs and stHIVs

| Template |  | Primer name | Primer sequence | Length(nt) | Product length (bp) |
| --- | --- | --- | --- | --- | --- |
| For Full-length SHIVs | | | | | |
| pSHIV_AD8-EO_ | For | D033 | GTCGACGAGACCATGGGTGGAGCTATTTCCATGAGGC | 37 | 10314 |
|  | Rev | D034 | GAATTCTACTTACTTGTTTGATGCAGAAGATGTATTAGCCTTAG | 44 |  |
| Z331MTF  Z331F 6  Z331F 13 | For | D035 | AAGTAAGTAGAATTCATGGATCCAGTAGATCCTAACCTA | 39 | 2981 |
|  | Rev | D036 | CATGGTCTCGTCGACTTATTGCAAAGCTGCTTCAAAGC | 38 |  |
| Z3618MTF  Z3618F 5 | For | D037 | AAGTAAGTAGAATTCATGGAGCCAATAGATCCTAAACT | 38 | 3034 |
|  | Rev | D038 | CATGGTCTCGTCGACTTATAGCAAAGCTACTTCAACAC | 38 |  |
| Z3618F 14 | For | D037 | AAGTAAGTAGAATTCATGGAGCCAATAGATCCTAAACT | 38 | 3010 |
|  | Rev | D039 | CATGGTCTCGTCGACTTATAGCAAAGCTCTTTCACAACC | 39 |  |
| Z3678MTF | For | D040 | AAGTAAGTAGAATTCATGGAGCCAGTAGATCCTAACCT | 38 | 2975 |
|  | Rev | D041 | CATGGTCTCGTCGACTTATAGCAAAGCTGCTTCAAAGCC | 39 |  |
| Z3678F 11 | For | D040 | AAGTAAGTAGAATTCATGGAGCCAGTAGATCCTAACCT | 38 | 2972 |
|  | Rev | D041 | CATGGTCTCGTCGACTTATAGCAAAGCTGCTTCAAAGCC | 39 |  |
| Z3678F 14 | For | D040 | AAGTAAGTAGAATTCATGGAGCCAGTAGATCCTAACCT | 38 | 2954 |
|  | Rev | D041 | CATGGTCTCGTCGACTTATAGCAAAGCTGCTTCAAAGCC | 39 |  |
| Z4248MTF | For | D042 | AAGTAAGTAGAATTCATGGACCCAGTAGATCCTAACCTAG | 40 | 3019 |
|  | Rev | D043 | CATGGTCTCGTCGACTTATTGCAAAGCTGCTTCAAGGCCC | 40 |  |
| Z4248F14 | For | D042 | AAGTAAGTAGAATTCATGGACCCAGTAGATCCTAACCTAG | 40 | 3022 |
|  | Rev | D043 | CATGGTCTCGTCGACTTATTGCAAAGCTGCTTCAAGGCCC | 40 |  |
| Z4248F 16 | For | D042 | AAGTAAGTAGAATTCATGGACCCAGTAGATCCTAACCTAG | 40 | 3040 |
|  | Rev | D041 | CATGGTCTCGTCGACTTATAGCAAAGCTGCTTCAAAGCC | 39 |  |
| For truncated SHIVs | | | | | |
| pSHIV_AD8-EO_ | For | D078 | ACCCACCTCCCAACCCCGAGGGGACCCGACAGGC | 34 | 10986 |
|  | Rev | D079 | CTACAGATCATCAACATCCCAAGGAGCATGATGC | 34 |  |
| Z331MTF | For | D080 | GTTGATGATCTGTAGTGTGATAGGGAACTTGTGGGT | 36 | 2044 |
|  | Rev | D087 | GGTTGGGAGGTGGGTCTGAAACGATAAAGGTGAG | 34 |  |
| Z331F 6 | For | D080 | GTTGATGATCTGTAGTGTGATAGGGAACTTGTGGGT | 36 | 2044 |
|  | Rev | D088 | GGTTGGGAGGTGGGTCTGAAACGACAAAGGTGAG | 34 |  |
| Z331F 13 | For | D081 | GTTGATGATCTGTAGTGTGCTAGGGAACTTGTGGGT | 36 | 2158 |
|  | Rev | D088 | GGTTGGGAGGTGGGTCTGAAACGACAAAGGTGAG | 34 |  |
| Z3618MTF | For | D082 | GTTGATGATCTGTAGTGGGATGGGGAACATGTGGGT | 36 | 2113 |
|  | Rev | D089 | GGTTGGGAGGTGGGTCTGAAACGACAGAGGTGAG | 34 |  |
| Z3618F 5 | For | D082 | GTTGATGATCTGTAGTGGGATGGGGAACATGTGGG | 35 | 2098 |
|  | Rev | D090 | GGTTGGGAGGTGGGTCTGAAACGAGAGAGGTGAG | 34 |  |
| Z3618F 14 | For | D083 | GTTGATGATCTGTAGTGGGATGGGGAACTTGTGGGT | 36 | 2089 |
|  | Rev | D089 | GGTTGGGAGGTGGGTCTGAAACGACAGAGGTGAG | 34 |  |
| Z3678MTF | For | D084 | GTTGATGATCTGTAGTGGGAGCTTGTGGGTCACAG | 35 | 2071 |
|  | Rev | D089 | GGTTGGGAGGTGGGTCTGAAACGACAGAGGTGAG | 34 |  |
| Z3678F 11 | For | D084 | GTTGATGATCTGTAGTGGGAGCTTGTGGGTCACAG | 35 | 2068 |
|  | Rev | D089 | GGTTGGGAGGTGGGTCTGAAACGACAGAGGTGAG | 34 |  |
| Z3678F 14 | For | D084 | GTTGATGATCTGTAGTGGGAGCTTGTGGGTCACAG | 35 | 2050 |
|  | Rev | D089 | GGTTGGGAGGTGGGTCTGAAACGACAGAGGTGAG | 34 |  |
| Z4248MTF | For | D085 | GTTGATGATCTGTAGTGTGAGAGGGAACTTGTGGGT | 36 | 2092 |
|  | Rev | D091 | GGTTGGGAGGTGGGTCTGGAATGACAAAGGTGAG | 34 |  |
| Z4248F14 | For | D085 | GTTGATGATCTGTAGTGTGAGAGGGAACTTGTGGGT | 36 | 2095 |
|  | Rev | D091 | GGTTGGGAGGTGGGTCTGGAATGACAAAGGTGAG | 34 |  |
| Z4248F 16 | For | D086 | GTTGATGATCTGTAGGGGAGTAGGGGACTTGTGGGT | 36 | 2113 |
|  | Rev | D091 | GGTTGGGAGGTGGGTCTGGAATGACAAAGGTGAG | 34 |  |
| For stHIVs | | | | | |
| Z331MTF | For | D099 | AAATCATTAAGGACTATGGAGGAGGAAAAGAGGTGGATAGCA | 42 | 8415 |
|  | Rev | D100 | GCTCCTCTAAAAGCTCCACTACCCATTCATCCCATGGTTC | 40 |  |
| Z331F 6 | For | D099 | AAATCATTAAGGACTATGGAGGAGGAAAAGAGGTGGATAGCA | 42 | 8415 |
|  | Rev | D100 | GCTCCTCTAAAAGCTCCACTACCCATTCATCCCATGGTTC | 40 |  |
| pSHIV_AD8-EO_ | For | D101 | AGCTTTTAGAGGAGCTCAAGCA | 22 | 905 |
|  | Rev | D102 | AGTCCTTAATGATTTTTACTTTCCTTCT | 28 |  |
