# Supplementary material for: High throughput generation and characterization of replication-competent clade C transmitter-founder simian human immunodeficiency viruses
Source: PLoS One. 2018 May 14;13(5):e0196942. doi: 10.1371/journal.pone.0196942 (PMC5951672; doi:10.1371/journal.pone.0196942)
Supplement: S3 Table — (DOCX) [file pone.0196942.s004.docx]

Table S3. Primers used for Env 375 mutant generation, Same set of forward and reverse primer was used for the mutant generation for Full-length and truncated SHIVs.

| Template |  | Primer | Primer sequence | Primer length(nt) |
| --- | --- | --- | --- | --- |
| For Env375 mutation (For Histidine H) | | | | |
| 1ZMTF.F  1ZMTF.t | For | D136 | CACATCACTTTAATTGTAGAGGGGAAT | 27 |
|  | Rev | D137 | AATTAAAGTGATGTGTTGTAATCTCTA | 27 |
| 2ZF6.F  2ZF6.t | For | D136 | CACATCACTTTAATTGTAGAGGGGAAT | 27 |
|  | Rev | D137 | AATTAAAGTGATGTGTTGTAATCTCTA | 27 |
| 3ZF13.F  3ZF13.t | For | D148 | CACACCACTTTAATTGTAGAGGGGAAT | 27 |
|  | Rev | D149 | ACAATTAAAGTGGTGTGTTGTAATTTCTA | 29 |
| 4ZMTF.F  4ZMTF.t | For | D140 | CACATCACTTTAATTGTAGAGGAGAAT | 27 |
|  | Rev | D142 | AATTAAAGTGATGTGTTGTAATTTCTA | 27 |
| 5ZF5.F  5ZF5.t | For | D140 | CACATCACTTTAATTGTAGAGGAGAAT | 27 |
|  | Rev | D144 | AATTAAAGTGATGTGTTGTGATTTCTA | 27 |
| 6ZF14.F  6ZF14.t | For | D140 | CACATCACTTTAATTGTAGAGGAGAAT | 27 |
|  | Rev | D142 | AATTAAAGTGATGTGTTGTAATTTCTA | 27 |
| 7ZMTF.F  7ZMTF.t | For | D163 | ACACATCATTTTAATTGTGGAGGAGA | 26 |
|  | Rev | D164 | ATTAAAATGATGTGTTGTAATTTCTA | 26 |
| 8ZF11.F  8ZF11.t | For | D163 | ACACATCATTTTAATTGTGGAGGAGA | 26 |
|  | Rev | D164 | ATTAAAATGATGTGTTGTAATTTCTA | 26 |
| 9ZF14.F  9ZF14.t | For | D167 | AGACATCATTTTAATTGTGGAGGAGA | 26 |
|  | Rev | D168 | ATTAAAATGATGTCTTGTAATTTCTA | 26 |
| 10ZMTF.F  10ZMTF.t | For | D140 | CACATCACTTTAATTGTAGAGGAGAAT | 27 |
|  | Rev | D142 | AATTAAAGTGATGTGTTGTAATTTCTA | 27 |
| 11ZF14.F  11ZF14.t | For | D140 | CACATCACTTTAATTGTAGAGGAGAAT | 27 |
|  | Rev | D142 | AATTAAAGTGATGTGTTGTAATTTCTA | 27 |
| 12ZF16.F  12ZF16.t | For | D140 | CACATCACTTTAATTGTAGAGGAGAAT | 27 |
|  | Rev | D146 | AATTAAAGTGATGTGTGGTGATTTCTA | 27 |
| For Env375 mutation (For Tyrosine Y) | | | | |
| 1ZMTF.F  1ZMTF.t | For | D138 | CACATTACTTTAATTGTAGAGGGGAAT | 27 |
|  | Rev | D139 | AATTAAAGTAATGTGTTGTAATCTCTA | 27 |
| 2ZF6.F  2ZF6.t | For | D138 | CACATTACTTTAATTGTAGAGGGGAAT | 27 |
|  | Rev | D139 | AATTAAAGTAATGTGTTGTAATCTCTA | 27 |
| 3ZF13.F  3ZF13.t | For | D150 | CACACTACTTTAATTGTAGAGGGGAAT | 27 |
|  | Rev | D151 | ACAATTAAAGTAGTGTGTTGTAATTTCTA | 29 |
| 4ZMTF.F  4ZMTF.t | For | D141 | CACATTACTTTAATTGTAGAGGAGAAT | 27 |
|  | Rev | D143 | AATTAAAGTAATGTGTTGTAATTTCTA | 27 |
| 5ZF5.F  5ZF5.t | For | D141 | CACATTACTTTAATTGTAGAGGAGAAT | 27 |
|  | Rev | D145 | AATTAAAGTAATGTGTTGTGATTTCTA | 27 |
| 6ZF14.F  6ZF14.t | For | D141 | CACATTACTTTAATTGTAGAGGAGAAT | 27 |
|  | Rev | D143 | AATTAAAGTAATGTGTTGTAATTTCTA | 27 |
| 7ZMTF.F  7ZMTF.t | For | D165 | ACACATTATTTTAATTGTGGAGGAGA | 26 |
|  | Rev | D166 | ATTAAAATAATGTGTTGTAATTTCTA | 26 |
| 8ZF11.F  8ZF11.t | For | D165 | ACACATTATTTTAATTGTGGAGGAGA | 26 |
|  | Rev | D166 | ATTAAAATAATGTGTTGTAATTTCTA | 26 |
| 9ZF14.F  9ZF14.t | For | D169 | AGACATTATTTTAATTGTGGAGGAGA | 26 |
|  | Rev | D168 | ATTAAAATGATGTCTTGTAATTTCTA | 26 |
| 10ZMTF.F  10ZMTF.t | For | D141 | CACATTACTTTAATTGTAGAGGAGAAT | 27 |
|  | Rev | D143 | AATTAAAGTAATGTGTTGTAATTTCTA | 27 |
| 11ZF14.F  11ZF14.t | For | D141 | CACATTACTTTAATTGTAGAGGAGAAT | 27 |
|  | Rev | D143 | AATTAAAGTAATGTGTTGTAATTTCTA | 27 |
| 12ZF16.F  12ZF16.t | For | D141 | CACATTACTTTAATTGTAGAGGAGAAT | 27 |
|  | Rev | D147 | AATTAAAGTAATGTGTGGTGATTTCTA | 27 |
